# A standardized postmortem protocol to assess the real burden of sudden infant death syndrome

**DOI:** 10.1007/s00428-020-02747-2

**Published:** 2020-01-23

**Authors:** Stefania Rizzo, Monica De Gaspari, Elisa Carturan, Beatrice Paradiso, Donata Favretto, Gaetano Thiene, Cristina Basso

**Affiliations:** 1 Cardiovascular Pathology Unit, Azienda Ospedaliera, Department of Cardiac, Thoracic, Vascular Sciences and Public Health, University of Padua Medical School, Via A. Gabelli, 61 35121 Padova, Italy; 2 Legal Medicine and Toxicology Unit, Azienda Ospedaliera, Department of Cardiac, Thoracic, Vascular Sciences and Public Health, University of Padua Medical School, Padova, Italy

**Keywords:** Sudden unexpected infant death, Sudden infant death syndrome, Autopsy, Molecular analysis, Inflammatory respiratory disease

## Abstract

Sudden unexpected infant death (SUID) is a major cause of death in infants < 1 year of age. Sudden infant death syndrome (SIDS) is a SUID still unexplained after post-mortem examination. In 2014, a protocol of post-mortem investigation was introduced to assess both the prevalence and the etiopathogenesis of SUID. Our aim was to compare SUID data before and after the application of a standardized autopsy protocol of investigation. In the time interval 2004–2018, SUID cases occurring in the Veneto Region, North-East Italy, were referred to our Core Lab. Since 2014, a complete autopsy was performed, including gross and histological study with toxicologic and molecular analysis carried out at the referral center. A total of 36 SUIDs (22 M, mean age 95.5 ± 80 days), 17 before (group A) and 19 after (group B) 2014, were collected. In group A, only 1 (6%) resulted as explained SUID, due to lymphocytic myocarditis and 16 (94%) were SIDS. In group B, 8 were SIDS (42%) and 11 (58%) explained SUID cases (*p* < 0.01), consisting of interstitial pneumonia and bronchiolitis in 9 and lymphocytic myocarditis in 2 cases. Molecular analysis was positive for viruses in 8 of them (73%). In conclusion, since the application of a standardized protocol of post-mortem investigation, inflammatory, mostly infective, cardio-pulmonary diseases have been identified as the most common cause of SUID, with SIDS falling from 94 to 42% of SUID. Efforts must be made to implement a uniform autopsy protocol to provide reliable epidemiological data on SIDS.

**Electronic supplementary material:**

The online version of this article (10.1007/s00428-020-02747-2) contains supplementary material, which is available to authorized users.

## Introduction

Sudden infant death syndrome (SIDS) is defined as the sudden unexpected death of an infant < 1 year of age, apparently occurring during sleep, when complete autopsy, review of the circumstances of death, and clinical history fail to identify a cause [1]. Sudden unexpected infant death (SUID) is a wider concept including SIDS, asphyxia from accidental suffocation, and ill-defined deaths for infants < 1 year of age [2]. Despite the major advances in scientific research and the implementation of preventive behaviors, SIDS is still one of the leading causes of post-natal and infant mortality in developed countries [3]. Although pathogenesis remains unclear, SIDS is generally accepted as a multifactorial disorder, with a triple risk hypothesis as the most credited one (including an intrinsic child vulnerability, a critical period in the development of the autonomic regulation of both the respiratory and the cardiovascular systems, and exogenous triggers) [4]. Several factors have been identified as associated with a higher incidence of SIDS such as male sex, prone sleeping, exposure to cigarette smoke, young age, specific ethnicities, family history of SIDS, and prematurity [5].

After the first brief report about genetic investigation in SIDS cases [6], the scientific interest grew rapidly leading to many studies addressing the role of pathogenic mutations in SIDS victims, although the real impact remains to be clearly proven. Particular attention was paid to mutations in genes coding for proteins of cardiac ion channels, which have been reported in up to 10% of SIDS, suggesting a potential (even though debated) role for neonatal genetic screening for the identification of mutations associated with cardiac conditions [7].

In 2006, the obligation of autopsy has been introduced for SUID investigation by the Ministry of Health in Italy and then a national protocol including molecular and toxicological analysis has been approved in 2014, with the aim to standardize autopsy practices, to improve diagnostic accuracy, and to provide research material on the subject [8, 9]. Genetic investigations were excluded from the protocol.

The aim of our study was to assess the real cause of death in a consecutive series of SUID cases analyzed at our referral Center of the Veneto Region, Italy, before and after the application at regional level of the national protocol for postmortem study of SUID.

## Material and methods

**Fig. 1:**
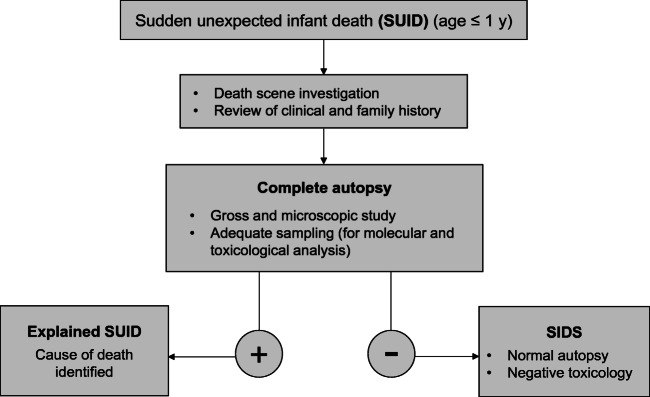
Flow chart of the mandatory national Italian protocol for SUID investigation

In the time interval January 2004 to December 2018, all SUID cases occurring in the Veneto Region, North-East Italy, were retrospectively reviewed. Death scene evaluation was always performed by the local forensic expert together with the police authorities: signs of violence, infant abuse or external agents’ involvement (carbon monoxide intoxication, signs of electrocution, or presence of fire in the room; signs of suffocation) were collected and, when positive, the case was excluded from referral to our center. The cases were distributed in two groups: one with the cases collected before the application of the standard national protocol (group A) and the other with those who died after the new law came into force and a regional Core Lab was identified (group B). For group A cases, autopsies including histological examination were performed at the local hospital and only the heart or the heart and lungs en bloc were sent to our unit for analysis. Since the application of the new protocol across the region in 2014, all SUID cases were obligatorily referred to our hospital and autopsy was performed according to a precise scheme (Fig. 1):

- Family history: SIDS or sudden death in other family members.
- Clinical history: history of fever or respiratory symptoms in the 5–7 days before death, any other condition reported since birth.
- Autopsy: all organs were examined, including the brain, heart, lungs, kidney, and adrenal glands, in order to find a cause of death. Following autopsy:(on a new line)
  - Extensive sampling of all organs for histological examination was performed for a total of 27 samples (see Supplemental file). Frozen samples of the cerebrospinal fluid, myocardium, lungs, and spleen (or blood) were taken for potential molecular evaluation.
  - Routine toxicological analysis was carried out in all SUID cases by looking for a primary or joint causal action of the most common sedatives or stimulants (alcohol, cocaine, opioids, and benzodiazepines).
  - Molecular analysis was performed in order to investigate the presence of viral pathogens. Viral DNA or RNA was searched directly in the frozen sample of the organs, taken during autopsy. A sample of blood was stored in EDTA.

The infants with known congenital conditions were excluded from the study.

After the complete postmortem investigation, the cases were identified as explained SUID (infants in which a certain cause of death was identified) or SIDS (SUID still unexplained after histological and toxicological examination), according to the general definition of SIDS by Krous [1]. The histological diagnosis of inflammatory diseases of the respiratory system, severe enough to explain the cause of death, was obtained according to the consensus on diagnostic criteria for the exclusion of SIDS [10]. In particular, interstitial pneumonia was diagnosed in the setting of widespread lymphoid infiltrates in the walls of the alveoli, bronchiolitis when lymphoid cells formed heavy cuffs around bronchi or bronchioles with obstruction and distal atelectasis. Myocarditis was diagnosed in the setting of myocyte necrosis, interstitial edema, and inflammatory cell infiltrates, with at least 2 foci showing CD3+ lymphocytes ≥ 7/mm^2^ [11].

### Histology/immunohistochemistry

Multiorgan autoptic tissue samples were fixed in 10% buffered formalin and paraffin-embedded; 5-μm-thick sections were stained with hematoxylin–eosin. For immunohistochemical studies, the following antibodies were used: anti-CD3 (ThermoFisher Scientific, 1:150), anti-CD20 (Dako, 1:100) and anti-CD68 (Dako, 1:50) to analyze the distribution and the lineage of inflammatory populations in the tissues. All antibodies were applied in a streptavidin biotin complex method with DAB as a substrate to visualize immunoreactivity.

### Molecular studies

PCR and reverse transcriptase RT-PCR were applied for detection of cardiotropic and respiratory viruses on the selected frozen tissues from autopsy: adenovirus, parainfluenza viruses 1 and 3, influenza virus A and B, respiratory syncytial virus (RSV), Epstein-Barr virus, cytomegalovirus (CMV), picornavirus-enterovirus/rhinovirus (EV/RV), herpes simplex virus, human herpes virus 6 (HHV6), and parvovirus B19. Total DNA and RNA were extracted and amplified and the results were analyzed according to the reported method [12, 13].

### Toxicological analysis

Toxicological analysis was performed at the Toxicology Unit by a general screening procedure, based on immunoassay of urine, liquid/liquid extraction of urine, blood, vitreous, bile, homogenization, and liquid/liquid extraction of tissues, followed by analysis of extracts by gas chromatography mass spectrometry (GC-MS) and liquid chromatography-high-resolution mass spectrometry (LC-HRMS) in full scan acquisition mode. Confirmation and quantification of identified drugs and metabolites are performed by Solid Phase extraction followed by LC-HRMS or LC-MS/MS.

### Statistical analysis

Data are expressed as mean ± SD. For statistical evaluation, results were analyzed by means of the Student’s *t* test. Differences were considered statistically significant at values of *p* < 0.05.

## Results

During the period 2004–2018, a total of 36 cases of SUID occurred in infants aged less than 1 year in the Veneto Region, Italy, and were collected at our unit. Data are reported in detail in Table 1. The 36 cases included 22 males and 14 females (age range 10–348 days, mean 95.5 ± 80 days, median 66.5 days). Death occurred during sleep in all cases, with infants found by parents in cardio-respiratory arrest. The forensic expert inspection excluded signs of violence and external agents’ involvement in the death of the infant for all referred cases.

**Table 1:** Clinical data and autopsy results in SUID victims before and after 2014, Veneto Region, Italy

Data	Before protocol	After protocol
SUID, n	17	19
Male sex, *n* (%)	12 (70.6)	10 (52.6)
Age, days, mean ± SD	99.4 ± 67.7	92.1 ± 91.4
Familial history positive for sudden cardiac death, *n* (%)	1 (5.9)	3 (15.8)
Caucasian ethnicity, *n* (%)	12 (70.6)	11 (57.9)
Death scene investigation		
Prone sleeping position, *n* (%)	NA	9 (47.4)
Co-sleeping, *n* (%)	NA	7 (36.8)
Clinical history		
Fever, *n* (%)	2 (11.8)	3 (15.8)
Respiratory symptoms, *n* (%)	1 (5.9)	5 (26.3)
Methodology		
Lungs examination at core lab, *n* (%)	9 (52.9)	19 (100)
Molecular test, *n* (%)	0 (0)	19 (100)
Toxicology, *n* (%)	17 (100)	19 (100)
Diagnosis		
SIDS, *n* (%)	16 (94.1)	8 (42.1)
Explained SUID, *n* (%)	1 (5.9)	11 (57.9)
Interstitial pneumonia/bronchiolitis, *n* (%)	0 (0)	9 (47.4)
Myocarditis, *n* (%)	1 (5.9)	2 (10.5)
Viral genome (in explained SUID)		
HHV6, *n* (%)	NA	4* (36.4)
RSV, *n* (%)	NA	3 (27.3)
EV, *n* (%)	NA	1* (9.1)
CMV, *n* (%)	NA	1 (9.1)
Seasonality of SIDS		
Winter, *n* (%)	7 (43.7)	5 (62.5)
Spring, *n* (%)	4 (25)	1 (12.5)
Summer, *n* (%)	2 (12.6)	0 (0)
Autumn, *n* (%)	3 (18.7)	2 (25)

*NA*, not available; *HHV6*, human herpes virus 6; *RSV*, respiratory syncytial virus; *EV*, enterovirus; *CMV*, cytomegalovirus; *1 patient double infection HHV6+EV

### Group A

Before the application of the standard national protocol, 17 SUID cases were referred to our unit. At histology, one case was diagnosed as lymphocytic myocarditis. Toxicological analysis was performed in all SUID cases and turned out negative. The remaining 16 infants (94%) showed no gross or histological lesions and were classified as SIDS.

### Group B

**Fig. 2:**
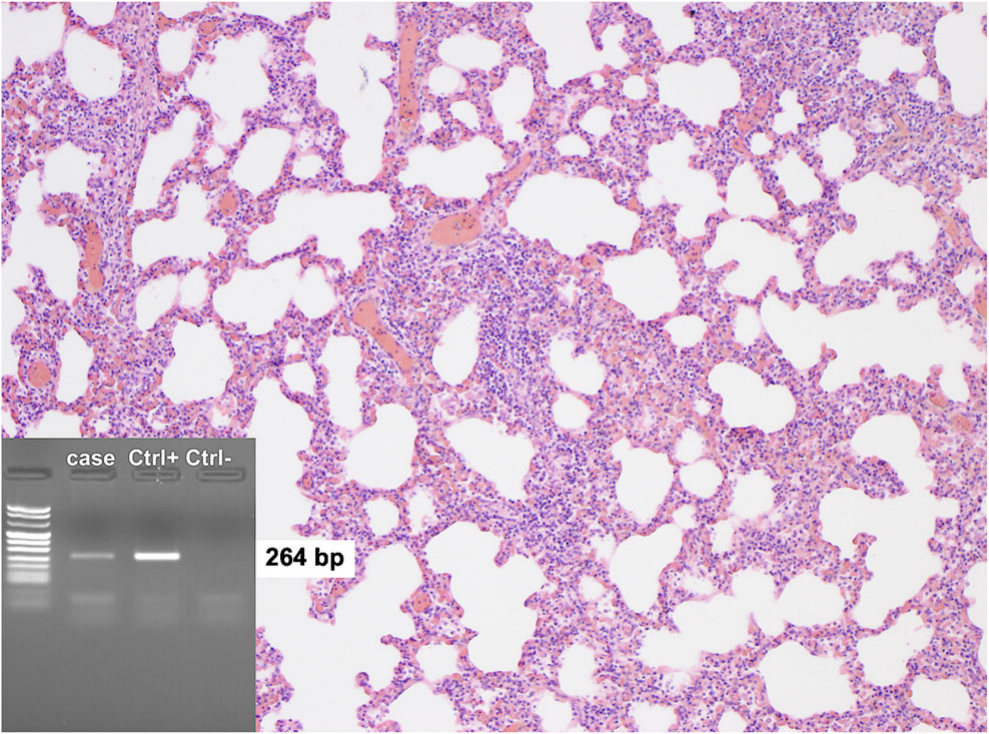
A 25-day-old male. Pulmonary histology demonstrated extensive lymphoid infiltrates in the walls of the alveoli. PCR was positive for HHV6 (H&E, original magnification × 50)

**Fig. 3:**
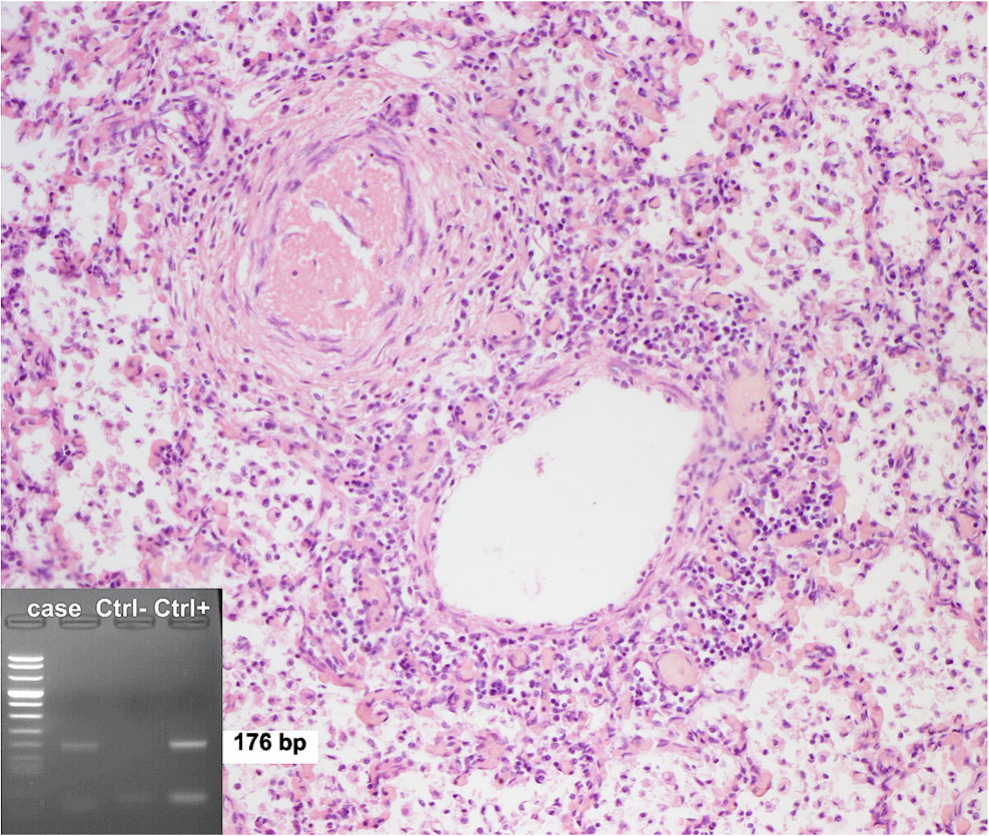
A 29-day-old male. Pulmonary histology showed massive infiltrates of lymphoid cells in the walls of bronchi causing obstruction due to bronchospasm. PCR was positive for respiratory syncytial virus (H&E, original magnification × 100)

**Fig. 4:**
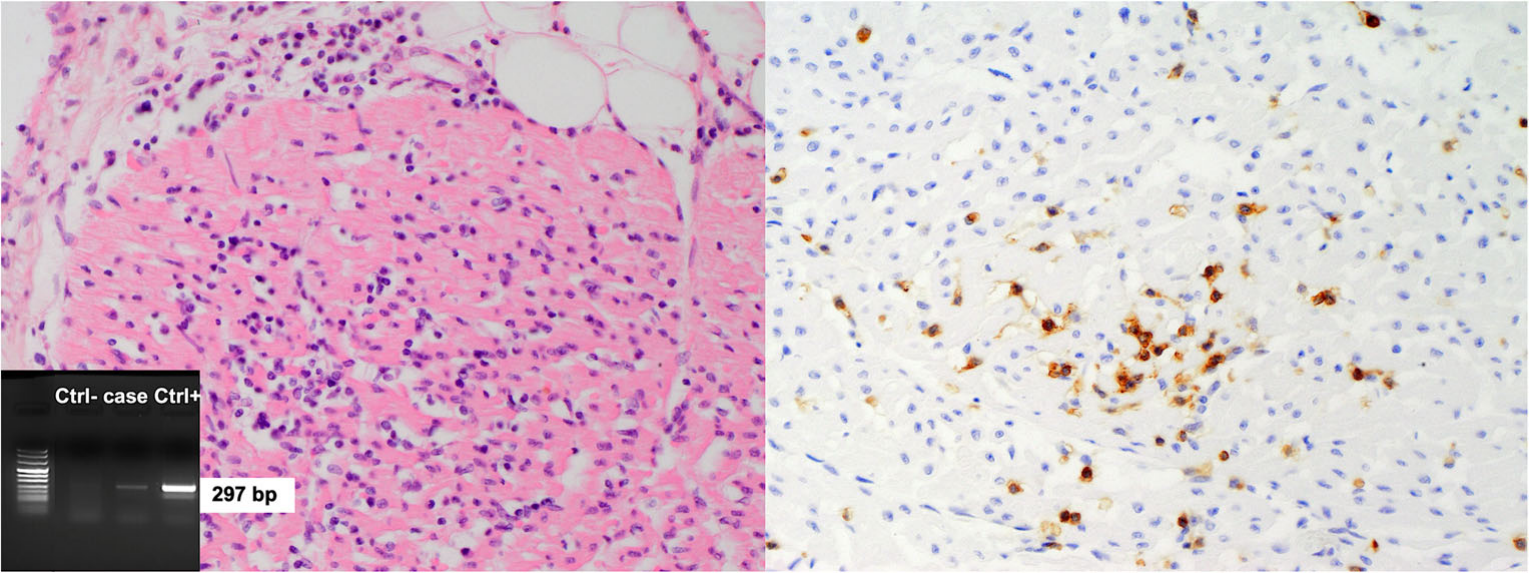
A 126-day-old, male. Histological examination of the anterior wall of the left ventricle revealed marked lymphocytic infiltration (CD3+) of the myocardium coupled with interstitial edema and myocyte necrosis. PCR was positive for enterovirus (H&E and immunohistochemistry for T lymphocytes CD3+, original magnification × 200)

**Fig. 5:**
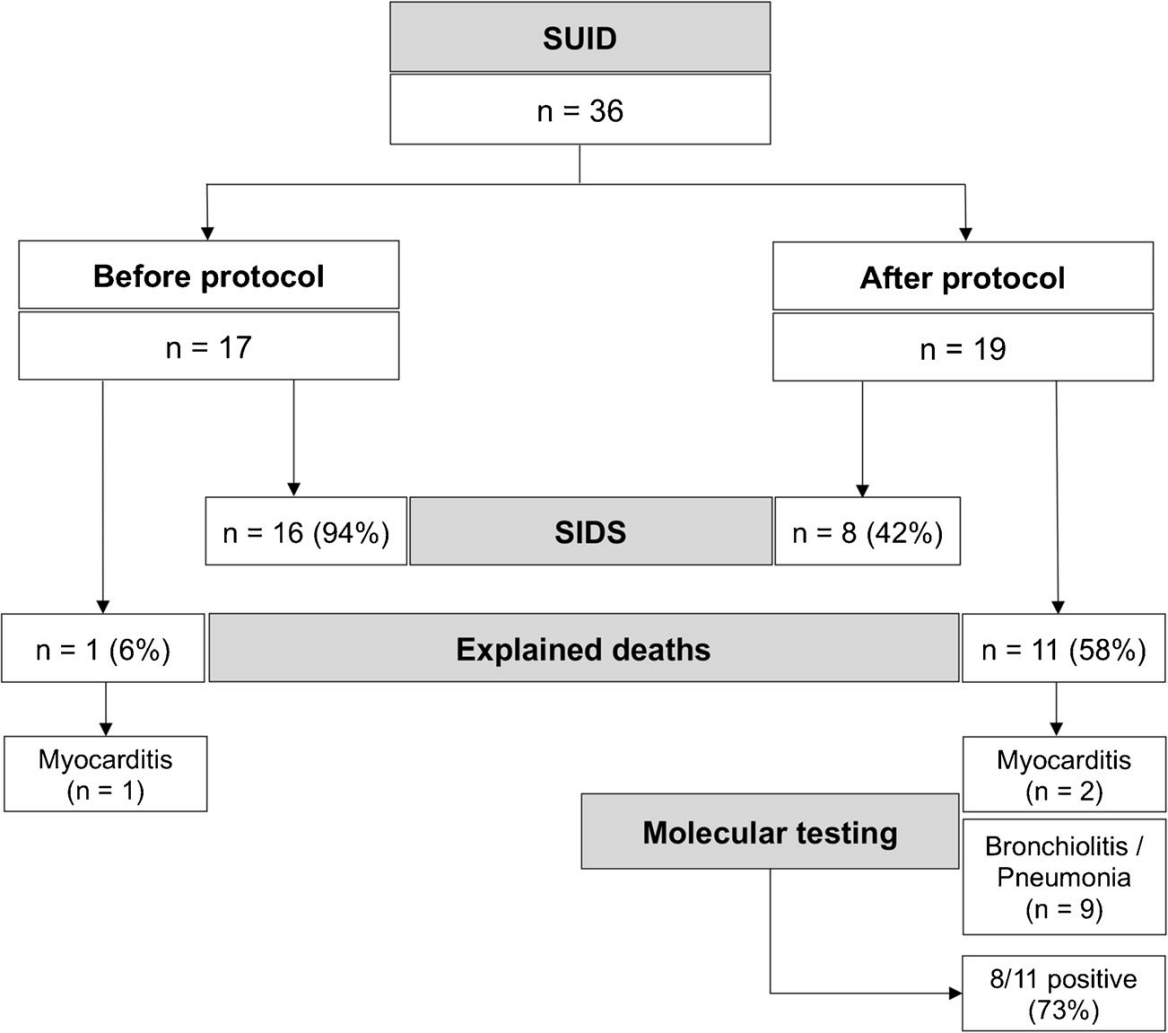
Explained SUID and unexplained SIDS cases variation before and after the introduction of the national protocol of post-mortem examination. Note the modification of the identified causes of explained SUID in the two groups

Since the new protocol application, 19 autopsies of SUID were performed. At autopsy, no external or internal abnormalities were found in all infants, except for petechiae in the thymus, pleura and pericardium, ungual cyanosis, and pulmonary edema. Histology demonstrated a cause of death in 11 explained SUID cases (58%). A virus, either in single or double infection, was identified in 8 out of 11 explained SUID (73%) vs in none of the 8 SIDS (Figs. 2, 3, and 4). Viral genome analysis in the blood was negative in all cases. All SUID cases underwent toxicological analysis and only one patient (7%) was positive for cocaine, but with a low non-toxic level. Cases in which a cause of death could not be identified were eventually classified as SIDS (8 infants corresponding to 42%). A schematic representation of the diagnostic results of both groups is reported in Fig. 5.

## Discussion

SIDS epidemiology is in constant evolution, mainly because of prevention campaigns and improvement in diagnostic techniques but also due to different classification systems [14]. Several US publications about SIDS investigation focused the attention on the death scenario reporting and the cause of death certification by medical examiners and coroners, because of different jurisdictional system types and subsequent ample variability in case investigation practices [15–17]. Supra-national comparison of SUID and SIDS rates is even more complicated by the use of different death certification codes and the results are highly variable [18, 19]. In general, the feeling is that the term SIDS has been overused and even applied to cases with obvious natural or unnatural causes of death.

The main finding of our investigation is the net decrease from 94 to 42% of the prevalence of SIDS in the SUID population, when a complete autopsy coupled with adequate sampling for histology is performed, according to a uniform standardized protocol, as required by the Italian law and then applied with the identification of a referral center at regional level in 2014. Noteworthy, histology performance rate is not constant among SIDS studies, varying from 60 to 95% [16, 17]. Our national protocol follows almost identically the international standardized protocol by Krous and Byard [20], in which a list of detailed macroscopic inspection and histological sampling is recommended.

We could presume that the remarkable higher rate of deadly respiratory infections in group B of our study could be ascribable to the availability of gross and histological examination of the infants’ airways, rather than consistent with a real decrease of SIDS. Consequently, we demonstrated that the performance of a complete autopsy with extensive histological sampling in all SUID cases can markedly change epidemiological data of SIDS.

In addition to the autopsy protocol, the absence of uniform diagnostic criteria at postmortem has been one of the major reasons for the large variations in SIDS rates throughout Europe and other countries, with explained SUID (non-SIDS) ranging from 2.5 up to 70% of cases. Thus, a consensus document was published to precisely classify histological findings in keeping with a certain cause of death (explained infant death) to allow the exclusion of SIDS [10]. According to that, our cases of explained SUID presented findings of undoubtedly fatal inflammatory disease, mostly infectious, in the upper and lower respiratory tract or myocardium. Since acquired airway infection/inflammation is frequent and children may not die suddenly, strict criteria have been adopted to provide a diagnosis of a causative disease (explained respiratory SUID), based upon histological changes at the level of alveoli, interstitial tissue, or bronchi and peri-bronchial tissue [10]. As far as myocarditis is concerned, we are well aware that the quantitative diagnostic criteria, which are routinely used in the pathology lab, are based upon the experience collected in living patients undergoing endomyocardial biopsy [11, 21, 22]. For this reason, in the current study, a postmortem diagnosis of myocarditis was provided only in the setting of at least two foci of clear-cut myocyte necrosis and inflammatory infiltrates with CD3+>7/mm^2^. An expert consensus document for the diagnosis of myocarditis at postmortem both in children and in the adult population is needed to avoid overdiagnosis at autopsy. Moreover, etiology of myocarditis is various, including not only infective (mostly viral) but also non-infective causes (toxic, immune, genetic predisposition, etc.) [11, 21–25]. When molecular pathology investigation was applied systematically in the setting of inflammatory cardio-respiratory diseases, such as interstitial pneumonia, bronchiolitis, and lymphocytic myocarditis, it provided additional data in terms of disease etiopathogenesis, by revealing viral genomes in three-quarter of SUID cases vs none of the histologically “negative” cases (SIDS), thus supporting a causative association.

Noteworthy, many studies addressed the role of bacterial and viral infection even in SIDS pathogenesis. The most plausible hypothesis is that common bacterial toxins, coupled with a viral infection, can be responsible for SIDS in a vulnerable infant [23]. This is consistent both with the seasonal peak incidence in winter, the age incidence (with a rise at 2–3 months when maternal IgG falls), and with the prone sleeping position (favoring accumulation of secretions and toxin production in upper airways). However, the actual low number of SIDS in the current series does not allow to collect significant data to draw any conclusion about SIDS pathogenesis. This is in fact out of the scope of our investigation, which is specifically addressing the real burden of SIDS among SUID, since SIDS has been possibly overestimated due to the use of incomplete postmortem protocols. Our results are anyhow in keeping with the higher prevalence of infants aged 2–4 months and the winter peak of SIDS cases, thus favoring the hypothetical role of infections in the complex etiological mechanisms.

The data here reported also confirm that the mechanism of death in SIDS may be either cardiac (rarely) or more frequently respiratory. The infants are found dead in the cot (“cot death”), with signs of asphyxia (cyanosis, pleural, or pericardial petechiae). The current scientific explanation of the respiratory mechanism of SIDS is an immaturity of vagal respiratory centers in the brain stem with prolonged pauses of the respiratory pacemaker, followed by asphyxia, bradycardia, and death. In other words, the primum movens is an incompetence of respiratory centers. The recent decrease of SIDS incidence by 50% was achieved by a simple maneuver, i.e., putting to sleep the infants in a supine (instead of prone) position, to facilitate ventilation. This supports a ventilatory contributing factor to hypoxia. The demonstration that a careful histological examination of the lungs with molecular analysis may reveal clinically occult inflammatory, often infective, disease, may further support a respiratory cause of death, by increasing the number of explained SUID.

According to the national protocol for SIDS investigation, molecular analysis should also include the search for mutations in genes encoding for inherited arrhythmic cardiac disorders and other non-cardiac disease-related genes such epilepsy. However, after a first period of great enthusiasm and interest due to the high prevalence of genetic variants, a more cautious approach is nowadays recommended to the application of genetic testing in SIDS [7]. Most of published studies do not involve the study of parents and/or family members, so that genotype-phenotype co-segregation analysis was not feasible and many previously published SIDS-associated mutations are probably now considered variants of unknown significance (VUS). While we are collecting blood samples in all SUID autopsies as the guidelines recommend, a comprehensive clinical evaluation of all available family members in the context of a multidisciplinary group is considered the *conditio sine qua non* prior to any further analysis, as recently suggested also for the study of sudden cardiac death in the young [26]. Prospective, systematic, case-control analysis with large SIDS cohorts and the use of uniform and rigorous methodology are required to confirm a role for genetic analysis in SIDS investigation [7, 27].

### Limitations of the study

As explained within the “Material and methods” section, signs of violence or external agents’ involvement were always searched for and when positive the case was excluded from referral to our pathology Core Lab. In other words, cases with unnatural causes of death such as asphyxia or abuse have never been referred to our Core Lab and we do not have the number of “excluded” cases. Our study addressed the natural SUID to better understand the real burden of SIDS by adopting a uniform pathology protocol in each case.

Finally, we recognize that group A (before protocol implementation) cannot directly be compared to group B (after protocol implementation). However, this is the starting point to say that an examination limited to few histological samples and without adopting a uniform protocol, including molecular virological investigation, is not sufficient to exclude natural structural causes of sudden death.

## Conclusion

Since the application of a rigorous autopsy protocol including histology of all organs and molecular test for SUID investigation, inflammatory, mostly infective, either respiratory or myocardial diseases have been the most common causes of “explained” SUID, while SIDS remained a diagnosis of exclusion accounting for less than half of cases. Efforts must be made to implement uniform autopsy protocols and diagnostic criteria to provide reliable epidemiological data on the phenomenon and to select real SIDS cases to be addressed to family investigation and molecular testing of inherited cardiac disease–associated genes, with important implications for cost, early diagnosis, and sudden death prevention.

## Electronic supplementary material

ESM 1 (DOCX 31 kb)
