# Supplementary material for: A standardized postmortem protocol to assess the real burden of sudden infant death syndrome
Source: Virchows Arch. 2020 Jan 23;477(2):177–83. doi: 10.1007/s00428-020-02747-2 (PMC7371652; doi:10.1007/s00428-020-02747-2)
Supplement: Supplementary file 1 — (DOCX 31 kb) [file 428_2020_2747_MOESM1_ESM.docx]

**Supplemental Material**

List of samples, Autopsy protocol Sudden Unexpected Infant Death, Veneto Region, Italy.

|  | **Organs to be stored (in 10% neutral buffered formalin)** |
| --- | --- |
| 1 | Heart and lungs *en bloc* |
| 2 | Brain stem |

|  | **Samples for histology (in 10% neutral buffered formalin)** |
| --- | --- |
|  | **Brain** |
| 1 | Cerebral hemispheres |
| 2 | Cerebellar hemispheres |
| 3 | Brain stem |
| 4 | Spinal cord |
| 5 | Meninges |
|  | **Lungs** |
| 6 | Right – superior lobe |
| 7 | Right – middle lobe |
| 8 | Right – inferior lobe |
| 9 | Left– superior lobe |
| 10 | Left – inferior lobe |
|  | **Heart** |
| 11 | Apical sample |
| 12 | **Thymus** |
| 13 | **Thyroid** |
| 14 | **Tongue - hypopharynx** |
|  | **Gastroenteric tract** |
| 15 | Esophagus |
| 16 | Stomach |
| 17 | Duodenum |
| 18 | Small bowel |
| 19 | Large bowel |
| 20 | **Right adrenal gland** |
| 21 | **Left adrenal gland** |
| 22 | **Right kidney** |
| 23 | **Left kidney** |
| 24 | **Spleen** |
| 25 | **Liver** |
| 26 | **Pancreas** |
| 27 | **Skeletal muscle (diaphragm or iliopsoas)** |

|  | **Samples for electron microscopy** | **Size** | **Storage** |
| --- | --- | --- | --- |
| 1 | **Heart** – ventricular myocardium | 1x1x1 mm | Karnovsky fixative at 4°C |

|  | **Samples for infective molecular diagnosis** | **Size** | **Storage** |
| --- | --- | --- | --- |
| 1 | **Cerebrospinal fluid** | > 1 ml | -20°C by 24 hours |
| 2 | **Heart** – apex | 5x5x5 mm | -80°C in aluminum foil |
| 3 | **Right and left lungs** | 10x5x5 mm | -80°C in aluminum foil |
| 4 | **Blood in EDTA and/or spleen** | > 1 ml – 3/5 g | -20°C by 24 hours |

|  | **Samples for genetic molecular diagnosis** | **Size** | **Storage** |
| --- | --- | --- | --- |
| 1 | **Blood in EDTA** | 5 ml | -20°C by 24 hours |
| 2 | **Spleen or Heart or Liver** | 5 g | -80°C in aluminum foil |

|  | **Samples for toxicological analysis** | **Size** | **Storage** |
| --- | --- | --- | --- |
|  | **Liquids** | | |
| 1 | **Cerebrospinal fluid** | *In toto* | -70°C < T < -20°C |
| 2 | **Vitreous humor** | *In toto* | -70°C < T < -20°C |
| 3 | **Heart blood (right ventricle)** | > 5 ml | -70°C < T < -20°C |
| 4 | **Peripheral blood (femoral vein)** | > 5 ml | -70°C < T < -20°C |
| 5 | **Gastric contents** | > 5 ml | -70°C < T < -20°C |
| 6 | **Duodenal contents** | > 5 ml | -70°C < T < -20°C |
| 7 | **Bile** | > 5 ml | -70°C < T < -20°C |
| 8 | **Urine** | > 5 ml | -70°C < T < -20°C |
|  | **Organ samples** | | |
| 9 | **Brain – frontal lobe** | > 5 g | -70°C < T < -20°C |
| 10 | **Lungs** | > 10 g | -70°C < T < -20°C |
| 11 | **Heart** | > 3 g | -70°C < T < -20°C |
| 12 | **Liver** | > 15 g | -70°C < T < -20°C |
| 13 | **Spleen** | > 5 g | -70°C < T < -20°C |
| 14 | **Kidneys** | > 5 g | -70°C < T < -20°C |
|  | **Tissues** | | |
| 15 | **Adipose tissue** | >10 g | -70°C < T < -20°C |
| 16 | **Skeletal muscle (iliopsoas)** | >15 g | -70°C < T < -20°C |
| 17 | **Hair (occipital region) – oriented sample** | *Total length* | -70°C < T < -20°C |

*Protocolli Diagnostici Nei Casi Della Morte Improvvisa Infantile e Della Morte Inaspettata Del Feto [Diagnostic Protocols in Cases of Sudden Infant Death and Unexpected Fetal Death]* (2014) Gazzetta Ufficiale della Repubblica Italiana, Italy: November 22; 2014:5-121.
